# Exercise, Epigenetics, and Body Composition: Molecular Connections

**DOI:** 10.3390/cells14191553

**Published:** 2025-10-06

**Authors:** Ashley Williams, Danielle D. Wadsworth, Thangiah Geetha

**Affiliations:** 1 Department of Food, Nutrition, and Packaging Sciences, Clemson University, Clemson, SC 29634, USA; 2 School of Kinesiology, Auburn University, Auburn, AL 36849, USA

**Keywords:** epigenetics, exercise, skeletal muscle, body composition, DNA methylation, histone modifications, non-coding RNAs

## Abstract

Exercise plays a crucial role in promoting overall health by activating molecular pathways that contribute to the prevention and management of chronic diseases, slowing epigenetic aging, improving body composition, and reducing the risk of obesity. In skeletal muscle, these benefits are largely mediated by exercise-induced transcriptional and epigenetic responses. Recent advances in epigenetics have intensified interest in understanding how physical activity influences long-term health and body composition at the molecular level. Epigenetic modifications, which regulate gene expression without altering the DNA sequence, are key mechanisms in this process. Emerging research has provided deeper insights into the processes such as DNA methylation, histone modification, and non-coding RNAs, and their connection to exercise. While numerous studies have demonstrated the influence of exercise on the epigenome, fewer have directly examined how these molecular changes relate to alterations in fat mass, lean body mass, and other components of body composition. This comprehensive review synthesizes the current evidence on the interplay between exercise, epigenetic regulation, and body composition, with a focus on adolescents and adults. We highlight key genes involved in metabolism, fat storage, muscle development, and epigenetic aging, and explore how their regulation may contribute to individual variability in exercise response. Understanding these molecular pathways may provide valuable insights for optimizing exercise interventions aimed at improving health outcomes across the lifespan.

## 1. Introduction

The effect of exercise on the human body has been extensively studied, with consistent and robust evidence demonstrating that regular physical activity significantly reduces the risk of major health conditions in children, adolescents, and adults [1]. These conditions include obesity, type 2 diabetes (T2DM), cardiovascular disease, and certain cancers [1]. In contrast, a sedentary lifestyle, especially when combined with poor dietary habits, is a major risk factor for noncommunicable diseases (NCDs), increased mortality, and other adverse health outcomes [2]. According to the World Health Organization (WHO), physical activity is defined as any bodily movement produced by skeletal muscles that require energy expenditure [3]. This includes a wide range of activities, such as household chores, walking, cycling, sports, and work-related activities. Regular participation in such activities is crucial for maintaining overall health and well-being. Exercise, a subset of physical activity, refers to planned, structured, repetitive, and purposeful movement aimed at improving or maintaining one or more components of physical fitness [4].

Exercise has a profound positive impact on human physiology, improving the efficiency of the respiratory and cardiovascular systems, strengthening the immune system, and enhancing neurophysiological function [5,6]. Among these systems, the cardiovascular system plays a central role, as increased heart rate and altered blood circulation are essential to meet the body’s increased oxygen demands during aerobic activity [7]. For adults, it is recommended to engage in at least 150 to 300 min of moderate-intensity endurance activity per week, or 75 to 150 min of vigorous exercise, or combination of both [8]. For children and adolescents, the guideline is at least 60 min of moderate to vigorous intensity exercise each day to achieve health benefits [9,10]. The WHO reports that insufficient physical activity increases the risk of death by 20% to 30% compared to adequately active individuals, making physical inactivity the fourth leading risk factor for global mortality [3,8]. In children and adolescents, insufficient exercise is often associated with poor muscular fitness and reduced motor skills, which reduces the likelihood of participating in sports or exercise, exacerbating poor muscular strength or motor coordination [11]. In addition to reducing the risk of chronic diseases, regular exercise plays a vital role in improving body composition, which is essential for overall health and longevity. Body composition assessment is a key tool for identifying and diagnosing various nutritional concerns, including overweight, obese, undernutrition, osteoporosis, sarcopenia, and sarcopenic obesity [12]. These assessments differentiate an individual’s total body mass into fat mass (FM) and lean body mass (LBM). FM consists primarily of adipose tissue, including both subcutaneous and visceral fat depots, with visceral fat being particularly associated with increased cardiometabolic risk due to its role in inflammation and metabolic dysregulation. LBM includes muscles, bones, organs, and other non-fat components [13]. Skeletal muscle is the largest component of LBM and is critical for mobility, strength, energy metabolism, and glucose regulation [14]. Bone mass provides structural support, protects vital organs, and acts as a reservoir for minerals; declines in bone mineral density are linked to conditions such as osteopenia and osteoporosis [14]. Additionally, organs such as the heart, liver, and kidneys contribute to LBM and overall physiological function despite constituting a smaller portion of total mass [15]. LBM is calculated as the difference between total body weight and FM. A healthy LBM typically accounts for 70–90% of total body weight, depending on age and gender, with men generally having a higher percentage than women [16,17,18]. FM, which consists primarily of adipose tissue, also varies by gender and age. For men, a healthy FM range is 12–20%, while for women, it is 20–30% of total body weight [12].

Several biological factors influence body composition, including age, gender, genetics, and race or ethnicity. Aging is naturally associated with increased FM accompanied by a gradual decline in LBM, particularly in bone and muscle [14,19]. Research consistently shows that women tend to have a higher percentage of body fat than men [20]. Excess body fatness has been linked to negative effects on physical fitness [21,22,23] and is linked to increased risk of cardiovascular diseases, T2DM, various cancers, and premature mortality [24]. Higher FM is also associated with poorer physical performance, including reduced cardiorespiratory fitness, lower scores on coordination and agility tests, weaker motor fitness and jumping ability, and lower self-esteem [25,26]. On the other hand, higher LBM, particularly muscle mass, has been positively correlated with greater handgrip strength and overall physical performance [15]. The association between body composition and physical fitness is linked to muscle metabolism, where increased energy expenditure effectively engages large muscle groups [15]. Since physical fitness is a key indicator of long-term health and tracks from childhood to adulthood [9], it is important to determine whether body composition serves as a significant and longitudinal predictor of other health-related physical fitness components.

Exercise not only influences body composition but also has the potential to reshape epigenetic markers, providing long-term health benefits. Obesity and related conditions are influenced by complex interactions between genetic predisposition, lifestyle behaviors, and genome–environment interactions, all which impact body mass, composition, and shape [27]. While genes play a fundamental role in health, lifestyle behaviors and environmental factors, such as diet and exercise, also significantly impact health outcomes. Epigenetics refers to changes in gene function that occur without altering the DNA sequence [28]. Unlike genetic mutations, which involve permanent alterations to the DNA sequence, epigenetic modifications are reversible and regulate gene expression [29]. Growing evidence suggests that environmental factors, including exercise, can induce rapid modifications in DNA methylation, thereby influencing gene expression [30,31]. Importantly, exercise and body composition can impact DNA methylation, potentially facilitating physiological adaptations to exercise and enhancing its health benefits [30,32]. For example, exercise-induced changes in DNA methylation regulate genes involved in metabolism, fat storage, muscle repair, and inflammation, which enhance the body’s ability to respond to physical activity [33]. The timing of these epigenetic modifications may be especially critical. Adolescence is a sensitive period for epigenetic reprogramming, during which many disease trajectories, such as those leading to obesity or metabolic disorders, are established [27]. However, despite increasing evidence supporting the role of epigenetics and body composition, the specific molecular mechanisms linking exercise, body composition, and epigenetic changes in both adults and adolescents remain underexplored. Body mass index (BMI), although widely used in clinical and public health settings, does not distinguish between FM and LBM. As a result, it may obscure important differences in metabolic and epigenetic profiles between individuals with similar BMIs [34]. Therefore, there is a pressing need to investigate how exercise-induced epigenetic changes relate to specific components of body composition, rather than relying solely on BMI.

This review focuses on the impact of exercise on body composition and epigenetic modifications, examining genes related to metabolism, fat storage, and muscle function. Additionally, it highlights key studies on how exercise and body composition influence epigenetic markers in both adults and adolescents. To identify pertinent human studies, a non-systematic search was conducted across electronic databases, including PubMed, Google Scholar, and Web of Science, utilizing search terms such as epigenetics, exercise, body composition, skeletal muscle, DNA methylation, gene expression, and epigenetic modifications. Both seminal and recent studies were considered to provide historical context and capture current advances. Efforts were made to reduce selection bias by including studies with varying methodologies and outcomes, including those reporting null or conflicting results. Nevertheless, as this is a narrative review rather than a systematic one, issues of bias and completeness cannot be fully eliminated.

## 2. The Role of Exercise in Body Composition

Exercise induces widespread gene expression changes across multiple organ systems, contributing to whole-body homeostasis and improved physical performance. Among these changes, skeletal muscle exhibits remarkable adaptability, rapidly responding to metabolic demands induced by different forms of exercise [31,32,33,35]. Understanding the molecular mechanisms driving these adaptations can guide more effective training strategies and optimize physical performance. In exercise physiology, two primary types of exercise are distinguished: aerobic (endurance) training and strength (resistance) training. Aerobic training typically consists of continuous, repetitive movements involving low resistance or force, placing primary demands on the cardiorespiratory system and primarily targeting the aerobic energy system, though anaerobic pathways may contribute at higher intensities [36]. In contrast, strength training utilizes higher loads with fewer repetitions, primarily targeting the neuromuscular system to enhance muscle strength, power, anaerobic endurance, and skeletal muscle size [36].

During exercise, motor units are recruited by the motor cortex, activating specific muscle fiber types depending on the exercise. There are different types of fibers: Type I fibers, which are fatigue-resistant and primarily use oxidative metabolism; Type IIx fibers, which are glycolytic, fast-twitch, and more prone to fatigue; and Type IIa fibers, which combine characteristics of both [6,37]. Simultaneously, exercise activates the cardiovascular, respiratory, endocrine, and metabolic systems, triggering key signaling pathways. These include adenosine monophosphate (AMP)-activated protein kinase (AMPK), protein kinase A (PKA), calcium/calmodulin-dependent protein kinase (CaMK), mitogen-activated protein kinase (MAPK), protein kinase C (PKC), and mammalian target of rapamycin (mTOR) [38]. AMPK, in particular, senses energy deficits and promotes mitochondrial biogenesis through activation of peroxisome proliferator-activated receptor gamma coactivator 1-alpha (PGC-1α) [31,39]. CaMK-II, another critical kinase, enhances glucose uptake through GLUT-4, supports lipid oxidation, and regulates transcription by modulating myocyte enhancer factor 2-histone deacetylase (MEF2-HDAC) complexes and transcription factors such as cyclic AMP response element-binding protein (CREB) and *MEF2* [40,41].

There are distinct molecular responses to endurance and resistance exercise. Resistance training typically activates the PI3K-Akt-mTOR signaling pathway, which regulates protein synthesis and degradation, leading to muscle hypertrophy and increased lean body mass [31,42]. In contrast, aerobic training primarily activates the AMPK-MAPK-PGC-1α pathway, resulting in increased mitochondrial biogenesis, enhance oxidative metabolism, a shift from fast glycolytic to more oxidative fiber phenotype and angiogenesis [33,42,43]. These molecular responses underline the physiological adaptations that influence body composition. Resistance training increases muscle mass and strength, elevating basal metabolic rate and promoting fat loss over time [43]. Meanwhile, endurance training enhances fat oxidation and energy efficiency, resulting in reduced FM and improved metabolic function [5,43]. Additionally, endurance training promotes a shift from glycolytic (fast-twitch) to oxidative (slow-twitch or fatigue resistance) muscle fiber types, further increasing mitochondrial content and reliance on fat as an energy source [44]. Together, these adaptations contribute to long-term improvements in health, performance, and body composition.

Although the relationship between exercise and body composition has been widely studied, much of the research has focused on a few common indicators, such as body fat percentage, body mass index (BMI), and waist circumference [45,46]. However, Dewi et al. (2021) found that fat mass percentage was a more accurate predictor of low physical fitness in obese individuals than BMI [47]. Their study demonstrated a stronger correlation between fat mass percentage and physical fitness, highlighting the need for more precise measures of body composition [47].

Biological sex differences have been observed in the relationship between exercise and body composition. Research has shown that in men, higher FM to FFM ratios were associated with lower physical activity levels, particularly in strength training and general sports engagement [20]. This suggests that increased FM may influence exercise patterns, potentially leading to higher BMI without necessarily indicating overweight status [20]. In contrast, in women, physical activity tends to reduce FM and increase muscle mass [24]. A 3-year longitudinal study in adolescent girls revealed that FM was inversely associated with fitness measures such as sit-ups and squats, while LBM was positively associated with performance [23]. Recent studies have explored alternative exercise methods, such as active video games (AVG), to promote fitness. A five-month intervention combining AVG with multicomponent training in overweight children showed significant improvements in lean mass and motor skills, which correlated with vigorous activity and jump performance [48]. AVGs may help engage children in less inclined toward conventional exercise by making physical activity more enjoyable [49].

## 3. Exercise and Epigenetic Regulation

### 3.1. DNA Methylation and Exercise

In recent years, extensive research has focused on the epigenetic changes induced by exercise and their physiological effects. The connection between exercise and epigenome, examining key epigenetic mechanisms such as DNA methylation, histone modifications, and non-coding RNAs [32]. Among these, DNA methylation is one of the most studied epigenetic mechanisms and plays a critical role in regulating gene expression.

DNA methylation is catalyzed by a highly conserved family of enzymes known as DNA methyltransferase (DNMT), which consists of five members: DNMT1, DNMT2, DNMT3A, DNMT3B, and DNMT3L. DNMTI maintains methylation patterns during DNA replication [50,51], while DNMT3TA and DNMT3B establish de novo methylation patterns [52]. DNA methylation typically occurs at CpG sites, where a cytosine is followed by a guanine nucleotide [53]. Approximately 70% of gene promoters reside within CpG islands, which are highly conserved and critical in regulating gene expression by altering chromatin structure and transcription factor accessibility [31,54,55,56]. While these enzymes regulate DNA methylation at specific loci, it is important to note that their activity is typically global across the genome. As such, although exercise can alter the expression or activity of DNMTs, it remains challenging to precisely identify which genes or pathways are functionally impacted without comprehensive epigenome-wide analysis.

Exercise has shown to induce DNA hypomethylation in key genes, serving as an early response mechanism to facilitate adaptation [31,57]. Muscle contraction triggers several cellular processes, including Ca^2+^ release and uptake, changes in AMP/ATP ratios that activate AMPK, and increased oxidative metabolism that generates reactive oxygen species (ROS) [57,58]. These processes influence the availability of S-adenosyl methionine (SAM), a methyl donor, thereby affecting DNA methylation patterns [58]. Thus, oxidative stress and calcium signaling are key regulators of exercise-induced DNA methylation [59].

Changes in skeletal muscle gene expression are crucial for exercise adaptations. Even a single bout of exercise can alter the muscle transcriptome [60,61]. Moreover, there is a significant difference in gene expression between trained and untrained skeletal muscles even at rest, particularly evident in endurance-trained individuals [62]. Although limited, studies have revealed that DNA methylation patterns shift rapidly following exercise [32] and vary based on intensity and duration [30]. A study found that participants aged 45–75 years who exercised 26–30 min per day exhibited significantly higher DNA methylation levels compared to sedentary individuals [63]. Additionally, endurance- and resistance-trained athletes demonstrate different DNA methylation patterns in skeletal muscle genes compared to untrained individuals [64]. Skeletal muscle fiber type distribution is also associated with the methylation of fiber type-specific genes. Slow-twitch (type I) fiber-associated genes, such as *MYH7* (encoding β-myosin heavy chain) and *MYL3* (myosin light chain 3), exhibit reduced promoter methylation and increased expression in endurance-trained individuals [32]. Increased expression of *MYH7* and *MYL3* due to hypomethylation likely promotes a shift toward a higher proportion of oxidative, fatigue-resistant slow-twitch fibers, enhancing endurance capacity, mitochondrial efficiency, and metabolic health [32]. This fiber type transition contributes to improved aerobic performance and metabolic flexibility, which are hallmarks of endurance training adaptations [32]. Elucidating the direct link between epigenetic regulation of fiber type-specific genes and functional changes in muscle fiber distribution is essential to fully understand how exercise shapes skeletal muscle phenotype and performance [32].

The most investigated genes in skeletal muscle adaptation include *PGC-1α*, pyruvate dehydrogenase kinase (*PDK4*), mitochondrial transcription factor A (*TFAM*), and myocyte enhancer factor 2A (*MEF2A*) [65]. *PGC-1α* regulates mitochondrial biogenesis, fatty acid oxidation, and insulin sensitivity, and is upregulated with exercise [66,67]. *PDK4*, associated with hyperglycemia, shows increased expression after both short-term high-intensity and long-duration low-intensity exercise [68]. Barres et al. (2012) demonstrated dose-dependent upregulation of *PGC-1α*, *PDK4*, and *PPAR-δ* with significant promoter hypomethylation, linking early hypomethylation to gene activation during muscle contraction [57]. *TFAM* plays a protective role against mitochondrial dysfunction and muscle atrophy [69]. In a mouse study, *TFAM* overexpression after a 2-week training period was associated with increased antioxidant expression and improved redox balance, suggesting its importance in muscle health [70]. *MEF2A*, a major regulator of *PGC-1α*, is crucial for muscle development [71]. Chronic aerobic exercise increases *MEF2A* and *PGC-1α* expression, promoting mitochondrial biogenesis and metabolic homeostasis [71].

While studies on skeletal muscle epigenetics in adolescents are limited, existing research highlights the influence of exercise on DNA methylation in youth. Most studies focus on obesity-related and growth-related genes. For example, in children aged 8–18 years, methylation levels of the Fas apoptotic inhibitory molecule 2 (*FAIM2*) promoter were significantly associated with sedentary behavior versus regular exercise [72]. *FAIM2*, a membrane protein expressed in the hippocampus, has been linked to obesity through genome-wide association studies [73]. Polymorphisms and promoter methylation near *FAIM2* may contribute to obesity risk [74]. In another study, replacing 30 min of sedentary behavior with vigorous exercise increased methylation of hydroxysteroid (11-beta) dehydrogenase 2 (*HSD11B2*) in 14.5-year-old boys [75]. *HSD11B2*, which regulates active cortisol levels in skeletal muscle, may influence inflammation [75,76]. Additionally, Woo et al. (2012) found that 12 weeks of exercise upregulated superoxide dismutase (*SOD*) and glutathione peroxidase (*GPX*) expression in overweight and normal-weight children [77]. These antioxidant genes help combat oxidative stress, improving cellular health [78].

Overall, the literature supports the consensus that exercise modulates DNA methylation in skeletal muscle, influencing genes involved in mitochondrial biogenesis, metabolism, fiber type specification, and antioxidant defense [57,65]. Hypomethylation appears to facilitate transcriptional activation of key metabolic and structural genes in both acute and chronic exercise [57,65]. However, contradictions exist in the magnitude and direction of methylation changes, likely stemming from differences in exercise modality, intensity, duration, population age, sex, and tissue sampling methods. Evidence in adolescents remains limited and often focuses on obesity- or growth-related genes, leaving a gap in understanding how developmental stage influences epigenetic responsiveness. Furthermore, while candidate gene studies highlight potential mechanisms, comprehensive epigenome-wide analysis is sparse, restricting our ability to map the broader network of exercise-responsive loci.

### 3.2. Histone Modification and Exercise

Compared to DNA methylation, histone modifications are more intricate as they occur on histone proteins, which are the primary components of chromatin responsible for packaging DNA into structural units called nucleosomes [7]. Histones are composed of amino acids with basic side chains, such as lysine and arginine residues, which are the main target of epigenetic modifications [7]. Each histone contains a histone fold domain, composed of three alpha helices connected by two loops, allowing heterodimeric interactions between core histones [79]. Additionally, each histone has an N-terminal domain called “histone tails” [79]. These histone tails are the primary sites for a variety of post-translational modifications, including acetylation, methylation, and phosphorylation, which are mediated by specific classes of enzymes [80,81]. These modifications modulate chromatin structure and function by altering the chemical properties of histones and regulating the recruitment of chromatin-associated proteins [80,81,82,83]. Thus, the combination of these modifications creates a complex and dynamic system that helps to shape gene expression patterns in response to developmental cues and environmental cues and exercise stimuli, such as exercise [31]. Exercise has shown to have a profound impact on histone marker distribution, highlighting its role in gene regulation.

Histone acetylation is a process, when an acetyl group from acetyl-CoA is transferred to a lysine residue on the histone tail, primarily catalyzed by histone acetyl transferases (HATs) [30,31]. This modification neutralizes the positive chargers on lysine side chains, weakening histone DNA interactions and resulting in a more open chromatin conformation [31]. As a result, DNA becomes more accessible to transcriptional machinery and regulatory proteins, thereby promoting gene activation While HAT activity promotes transcriptional activation, HDAC activity facilitates chromatin condensation and transcriptional repression. While these enzymes regulate histone modifications broadly across the genome, their activity affects many loci simultaneously [84,85]. This global activity makes it challenging to determine which specific gene targets or physiological processes, such as those related to exercise adaptation, are directly affected without high-resolution, locus-specific analysis. Therefore, although exercise-induced shifts in HAT or HDAC activity correlate with chromatin remodeling and gene activation, pinpointing the precise genes or pathways directly influenced by these global changes remains challenging without high-resolution, genome-wide analyses [85]. This complexity underscores the need for integrative studies combining epigenomic mapping with transcriptomic data to clarify how exercise modulates specific gene networks via histone modifications [84,85]. Exercise has been associated with the histone acetylation in human skeletal muscle, correlating with chromatin decompaction and the activation of transcription of exercise responsive genes. Lim et al. (2020) showed that resistance training leads to an upregulation of acetylated H3 [86]. McGee et al. (2009) demonstrated that a single bout of exercise H3K36 acetylation, which may be associated with enhanced transcription elongation of certain exercise associated genes [87]. These researchers also observed increased nuclear-to-cytosolic translocation of HDACs 4 and 5 during exercise, which contributes to enhanced H3 acetylation [87,88]. Furthermore, the dissociation of HDACs 4 and 5 from the myogenic transcription factor MEF2 (myocyte enhancer factor 2) enhanced DNA binding of MEF2 [89,90].

Histone methylation, which can occur on both lysine and arginine residues, is catalyzed by histone methyltransferases (HMTs) and removed by histone demethylases (HDMs) [91]. Unlike acetylation, methylation does not alter the charge of the histone but instead serves as a specific docking site for effector proteins containing recognition domains such as chromodomains, Tudor domains, or PHD fingers [83]. The functional outcome of methylation is highly context specific as the tri-methylation of histone H3 at lysine 4 (H3K4me3) is typically associated with active transcription, while H3K9me3 and H3K27me3 are hallmarks of transcriptional repression and heterochromatin formation [92]. Histone methylation, particularly on histones H3 and H4, has also been shown to respond to physiological stimuli such as exercise [91]. For example, a single session of aerobic training can affect the levels of HDACs in obese individuals to reduce inflammation [93]. Additionally, a single session of combined aerobic and resistance exercise can alter the level of histone H4 acetylation and cytokine production patterns in schizophrenia patients [94]. H3K4 methylation, which is highly abundant in promoter regions and transcriptional start sites, has been found to increase with exercise [31,93] indicating its role in transcriptional activation.

Other histone modifications, such as phosphorylation of serine and threonine residues (e.g., H3S10ph), play important roles in processes such as chromosome condensation during mitosis and the DNA damage response [95]. Histone phosphorylation typically occurs at serine, threonine, and tyrosine residues in the N-terminal histone tails and is regulated by kinases and phosphatases [83]. Yu et al. (2003) reported a significant increase in H3 phosphorylation following an intense cycling exercise protocol consisting of eight 5 min bouts at ~85% of VO_2_peak, with 60 s recovery periods between each bout [96]. Several signaling pathways, including AMPK, MAPK, PKA, PKC, and CaMK-II, are involved in phosphorylation-dependent signaling during exercise in skeletal muscle [33,42]. Notably, both AMPK and CaMK-II have been shown to directly phosphorylate H3 [97]. Overall, histone modifications act in a combinatorial and dynamic manner, interpreted by specific reader proteins that initiate downstream biological processes such as transcription, DNA repair, or replication, thus serving as a sophisticated regulatory interface between the genome and the epigenome. Both acute and chronic exercise influences these modifications in skeletal muscle, contributing to adaptions in metabolism, inflammation, mitochondrial function, and muscle phenotype [87,90]. However, the specific loci affected by exercise, the differential responses to varying exercise modalities, and the interplay between multiple histone marks are not fully understood. Most studies focus on adults, leaving adolescent populations largely unexplored. Integrative, genome-wide studies combining epigenomic and transcriptomic analyses are needed to clarify the precise role of histone modifications in exercise-induced adaptations.

### 3.3. MicroRNAs (miRNAs) and Exercise

Non-coding RNAs, including microRNAs (miRNAs), represent a critical layer of gene regulation that operates independently of protein-coding function [98]. Like histone modifications, miRNAs primarily exert control through post-transcriptional mechanisms, enabling fine-tuned control over gene expression [98]. Initially, miRNAs are transcribed as primary miRNAs (pri-miRNAs) by RNA polymerase II [99]. These are processed in the nucleus by the Drosha-DGCR8 complex into precursor miRNAs (pre-miRNAs), which have a characteristic hairpin structure [99]. The pre-miRNAs are then exported to the cytoplasm via Exportin-5 and further cleavage by the Dicer enzyme, producing mature double-stranded miRNAs [99]. One strand (the guide strand) is incorporated into the RNA-induced silencing complex (RISC), while the passenger strand is typically degraded [99]. The resulting RISC–miRNA complex binds target mRNAs via partial complementarity, usually within the 3′ untranslated region (3′ UTR), leading to either translational repression or mRNA degradation [98]. A single miRNA can regulate hundreds of different transcripts, often targeting multiple genes within the same biological pathway [100]. Similarly to DNA and histone modification enzymes, many miRNAs exert broad regulatory effects across multiple genes and pathways, making it challenging to pinpoint the exact downstream processes or targets specifically responsible for exercise-induced changes in body composition. This allows miRNAs to exert broad regulatory influence over processes such as cell proliferation, apoptosis, differentiation, and metabolic adaptation [100]. Accordingly, they are key regulatory nodes in both physiological and stress-responsive pathways.

In the context of exercise, miRNAs modulate mitochondrial metabolism, inflammation, muscle recovery, and hypertrophy in skeletal muscle [101,102]. Identifying exercise-induced miRNA expression patterns could provide valuable biomarkers for monitoring physical fatigue, recovery, and overall performance capacity [7,103]. MiRNAs are short, non-coding RNA molecules, typically 18–24 nucleotides in length, that function as post-transcriptional regulators of messenger RNA (mRNA), playing essential roles in RNA silencing and gene expression regulation [104,105,106]. Their presence is often linked to non-homeostatic conditions, which makes them especially relevant to exercise physiology [1]. Several miRNAs are known to regulate signaling pathways critical to exercise adaptation [107].

MiRNAs function in a tissue-specific manner, and those expressed exclusively in skeletal muscle are referred to as myomiRs. Currently, seven myomiRs associated with skeletal muscle have been identified: miR-1, miR-133a, miR-133b, miR-206, miR-208b, miR-486, and miR-499 [103,105,108]. These myomiRs are involved in controlling muscle biogenesis, regeneration, fiber-type specification, and the maintenance of skeletal muscle homeostasis [109,110,111]. Their expression is regulated by both the type and duration of exercise. Supporting this, Russell et al. (2013) demonstrated that a single session of moderate-intensity endurance cycling increased expression of miR-1, miR-133a, and miR-133b in skeletal muscle, while the levels of other miRNAs were downregulated, suggesting a role in the early response to exercise-induced muscle stress and adaptation [111]. In contrast, Nielsen et al. (2010) found that chronic endurance exercise (12 weeks) led to downregulation of miR-1, miR-133a, miR-133b, and miR-206 in the human vastus lateralis [112]. This downregulation was associated with improved endurance capacity, VO_2_max, and insulin sensitivity, indicating a potential role for reduced myomiR expression in long-term metabolic and performance adaptations [112]. Together, these findings suggest that myomiRs may exert distinct roles during the acute and chronic phases of exercise adaptation.

Although research on miRNAs in youth is limited, a study by Virens et al. (2018) reported that increased weekly screen time was significantly associated with elevated salivary extracellular levels of miR-146a and miR-222 [113]. Specifically, each additional hour of weekly screen time was linked to a 3.44% increase in miR-222 and a 1.84% increase in miR-146a expression [113]. These circulating miRNAs are involved in inflammatory responses and have been implicated in diseases such as cancer and cardiovascular disease [114]. However, no significant associations were found between exercise, BMI, and miRNA expression.

While challenges remain in detecting circulating miRNAs due to the lack of standardized protocols to reduce methodological bias, several tissue-specific miRNAs are released into circulation in response to exercise-induced physiological stimuli [104]. Their expression appears to be influenced by the type and intensity of exercise. A summary of key studies investigating the epigenetic impacts of exercise, including DNA methylation, histone modifications, and miRNA regulation is presented in Table 1.

Overall, exercise modulates miRNA expression in skeletal muscle and circulation, influencing pathways related to muscle growth, regeneration, fiber-type specification, metabolism, inflammation, and recovery [111,112]. Acute and chronic exercise appear to induce distinct miRNA responses, with some miRNAs upregulated during early adaptation and downregulated following long-term training, reflecting dynamic regulation of muscle remodeling and metabolic function [111,112]. Future high-resolution, longitudinal studies integrating miRNA profiling with transcriptomic and functional outcomes are essential to clarify their precise contributions to exercise adaptations.

## 4. The Interaction Between Body Composition and Epigenetic Regulation

### 4.1. Fat Mass 

FM is a primary component of body composition influenced by both genetic and lifestyle factors. Exercise promotes FM reduction by enhanced energy expenditure and lipid metabolism [115]. Emerging evidence indicates that these effects may be mediated, at least in part, by epigenetic mechanisms. Moleres et al. (2013) conducted a 10-week lifestyle weight loss intervention in overweight and obese adolescents, categorizing participants as high or low responders [116]. They identified five DNA regions with different methylation patterns depending on weight loss response. Based on these findings, researchers developed an epigenetic score, calculated from baseline gene methylation differences between high and low responders, to evaluate the weight loss outcomes in a multidisciplinary program. These methylation changes offer deeper insight into personalized approaches for reducing FM and increasing LBM in adolescents [116]. Similarly, Keller et al. (2016) identified DNA methylation signatures associated with body weight loss after an 18-month lifestyle intervention, demonstrating that sustained behavioral changes can lead to lasting epigenetic modifications [102]. As research into the interplay between epigenetics and body composition expands, epigenome-wide association studies (EWAS) have begun identifying novel biomarkers. Tian et al. (2023) conducted an EWAS in monozygotic twins and identified 32, 22, and 28 differentially methylated CpG sites, and 20, 17, and 8 differentially methylated regions, associated with body fat percentage, FM, and LBM, respectively [34]. These epigenetic markers mapped to 65 genes, including well-known obesity-related genes such as PPARs, *DUSP4*, and *BAIAP3*. Peroxisome proliferator-activated receptors (PPARs), primarily expressed in the liver and adipose tissue, regulate fat metabolism, fatty acid oxidation, and energy homoeostasis [117]. Dual-specificity phosphatase 4 (*DUSP4*), a mitogen-active protein kinases (MAPKs) regulator, plays a role in metabolic homeostasis and is upregulated in liver tissue of individuals with obesity [118]. BAI1 associated protein 3 (*BAIAP3*) has been linked to regulation of circadian rhythms of food intake and energy balance [119].

Of particular interest is *PPAR-δ*, a key regulator of fatty acid oxidation. Acute exercise induces hypomethylation of *PPAR-δ* in skeletal muscle, leading to increased gene expression [57]. Although much research focuses on skeletal muscle, these findings indirectly suggest that exercise-induced epigenetic changes contribute to FM reduction. Direct evidence is provided by Rönn et al. (2013) who conducted a six-month exercise intervention and examined genome-wide DNA methylation patterns in human adipose tissue [120]. Their findings revealed significant methylation changes in genes regulating metabolic processes, such as *TCF7L2* (6 CpG sites) and *KCNQ1* (10 CpG sites) demonstrating that sustained exercise can alter the epigenetic landscape of fat storage tissue [120]. This provides direct evidence that long-term exercise can induce functional epigenetic changes in adipose tissue, offering a plausible mechanism for FM reduction through lifestyle interventions.

### 4.2. Lean Body Mass and Skeletal Muscle

**Figure 1:**
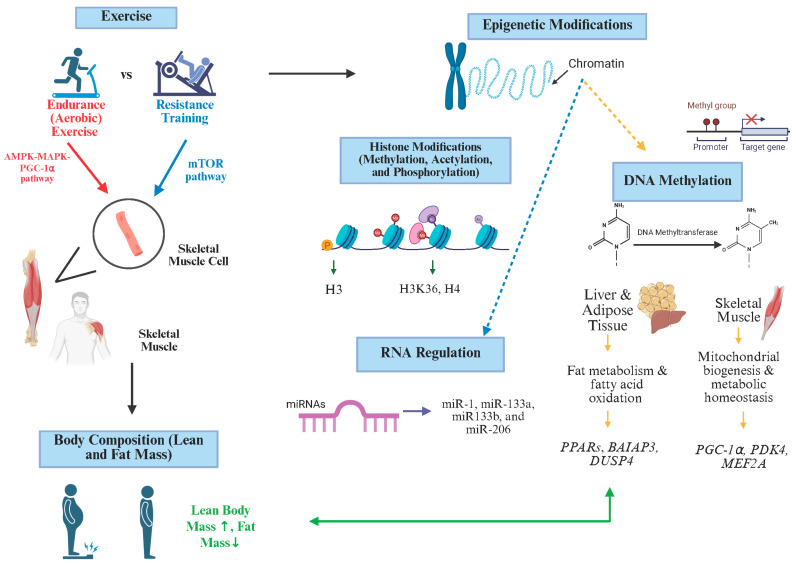
This figure illustrates the multifaceted relationships between exercise, body composition, and epigenetic changes. Exercise influences gene expression, leading to improvements in body composition, such as increased lean body mass and reduced fat mass. In turn, changes in body composition, particularly increased muscle mass, can further alter gene expression, promoting additional adaptations in physical performance and metabolism. Abbreviations; mTOR, mammalian target of rapamycin; AMPK-MAPK-PGC-1α, adenosine monophosphate activated protein kinase–mitogen activated protein kinase–peroxisome proliferator activated receptor gamma coactivator 1 alpha; *PDK4*, Pyruvate dehydrogenase kinase; PPARs, Peroxisome proliferator activated receptor; *DUSP4*, Dual-specificity phosphatase 4; *BAIAP3*, BAI1 associated protein 3; *MEF2A*, Myocyte enhancer factor 2A; H3, Histone H3 acetylation/phosphorylation; H3K36, Histone H3K36 acetylation; H4, Histone H4, acetylation.1. Created in https://BioRender.com accessed on 12 August 2025.

Aging, combined with lifestyle factors such as diet and physical activity, contributes to genetic and epigenetic modifications that influence body composition, including muscle mass maintenance and fat distribution (Figure 1). LBM, specifically skeletal muscle, plays a central role in metabolic regulation and insulin sensitivity [121]. Exercise, particularly resistance and endurance training, is well known to improve muscle mass [36]. Epigenetically, these improvements may involve exercise-responsive genes that regulate muscle remodeling, mitochondrial function, and glucose uptake.

Tian et al. (2023) reported that 28 CpG sites and 8 methylated regions were associated with LBM, including methylation changes in PPARs, *DUSP4*, and *BAIAP3* [34]. While PPARs are linked to muscle oxidative capacity, *DUSP4* functions as a negative regulator of the MAPK pathway and is associated with insulin signaling and inflammation [117,118]. However, no studies to date have directly shown exercise induced changes in *DUSP4* methylation.

In contrast, *NR4A1* has consistently demonstrated exercise responsiveness. For example, Maasar et al. (2021) reported hypomethylation of the *NR4A1* promoter and increased gene expression in human skeletal muscle following acute sprint exercise [122]. *NR4A1* appears to regulate glucose metabolism and mitochondrial function for high intensity aerobic training. Supporting this, Kasch et al. (2018) showed that maternal high fat feeding (40% energy from fat, 23% from protein, 37% from carbohydrates) altered *NR4A1* promoter methylation in offspring skeletal muscle, but voluntary exercise normalized methylation levels and improved insulin sensitivity, suggesting potential intergenerational benefits of physical activity [123].

Although Keller et al. (2016) did not differentiate FM and LBM in their outcome measures, the DNA methylation differences they observed between active and sedentary individuals suggest that changes in lean tissue may also be epigenetically regulated in the context of long-term exercise [102].

### 4.3. Bone and Other Components

Compared to fat and muscle, the epigenetic effects of exercise on bone tissue are not well characterized. Mechanical loading from resistance training promotes bone remodeling, and pathways such as Wnt/β-Catenin signaling are known to regulate bone mass [124,125]. While some evidence suggests that these pathways are epigenetically regulated, few studies have directly linked exercise-induced methylation changes to bone composition.

Given that bones are a significant part of total LBM, especially in adolescence and aging, further research is needed to explore whether physical activity can induce epigenetic changes in osteogenic genes and how these changes influence bone health outcomes.

Collectively, current evidence indicates that exercise can induce epigenetic modifications in genes regulating fat metabolism, muscle development, and possibly bone remodeling. However, the field remains in its early stages, especially with respect to establishing direct causal links between methylation changes and alterations in FM, LBM, or bone composition. Tissue-specific and longitudinal studies are needed to elucidate these mechanisms and translate findings into personalized health interventions.

## 5. Limitations, Contradictions, and Future Directions 

Several limitations and contradictions were identified in the reviewed studies on exercise-induced epigenetic regulation and its impact on body composition. First, inconsistencies in methodology across research articles pose challenges in drawing clear conclusions. For instance, differences in exercise type (endurance vs. strength), intensity, duration, variations in genes studied, and sample collection methods (muscle biopsies vs. blood samples) complicate cross-study comparisons. Moreover, genetic predispositions, age, and baselines of fitness levels among diverse participant groups could significantly impact methylation patterns due to population-specific factors. Additionally, the insufficient consideration of nutrition, particularly fasting periods prior to exercise interventions, may have epigenetic implications.

Contradictory findings are evident regarding the direction and magnitude of epigenetic changes. For example, some studies report exercise-induced hypomethylation of metabolic and structural genes, including *PGC-1α*, while others show modest or locus-specific effects. Acute vs. chronic exercise may differentially affect histone modifications and miRNA expression, with some miRNAs upregulated in early adaptation but downregulated following long-term training. These inconsistencies reflect the complex interplay between exercise modality, participant characteristics, tissue specificity, and study design. Mechanistic uncertainty further complicates interpretation. The same exercise stimulus may elicit different epigenetic outcomes depending on intracellular signaling, fiber-type composition, or redox status. For instance, calcium signaling, AMPK activation, and oxidative stress modulate DNA methylation and histone modifications, yet these processes vary across individuals and muscle types.

Another major limitation is the lack of studies involving adolescents in the context of exercise and epigenetics. Furthermore, research on sex differences in response to exercise and body composition remains limited. While it is generally observed that women have lower LBM and higher FM than men, this area remains underexplored. Given that exercise is a powerful modulator of gene expression via epigenetic mechanisms, these differences warrant further investigation.

In the context of body composition, significant gaps remain in our understanding, as many researchers focus on a few common indicators, such as body fat percentage, BMI, and waist circumference. This approach overlooks the important aspects of body composition, such as LBM and FM, both of which are significantly influenced by exercise. Overall, these contradictions and gaps highlight the need for more integrative, mechanistically informed research on the complex relationships between exercise, body composition, and epigenetics. Future research is needed to explore the direction of these associations whether casual, reversed, or bidirectional by utilizing more experimental or longitudinal studies. Such research is essential to deepen our understanding of how exercise influences body composition and epigenetic regulation in both adolescents and adults.

## 6. Conclusions

This comprehensive review explores the influence of exercise on body composition and epigenetic modification across diverse genes. Current literature provides strong evidence that gene expression and epigenetic modifications play a crucial role in regulating the body’s response to environmental stimuli, such as exercise, which in turn impacts body composition. These modifications are particularly evident in processes such as DNA methylation, histone modification, and microRNA regulation. Emerging research on epigenetic aging further highlights the connection between these molecular changes and the development of chronic diseases, morbidity, and mortality. To gain a better understanding of the epigenetic impact of different types of exercise, durations, and their impact on body composition, it is crucial to explore the multiple network mechanisms behind these processes, which can help identify biological factors that influence aging and health outcomes. Furthermore, elucidating the importance of exercise and body composition on epigenetic modifications helps to promote the development of therapeutic approaches and develop attainable tools to manage metabolic disease and potentially slow the progression of age-related conditions.

## Figures and Tables

**Table 1:** Summary of Studies on the Relation Between Physical Activity and Epigenetics.

				Human Studies			
Gene/ Marker	Modification Type	Function	Tissue/ Region	Exercise Protocol & Subjects	Epigenetic & Gene Expression Changes	Physiological Consequences	References
*PGC-1α*	DNA Methylation	Regulates mitochondrial biogenesis, fatty acid oxidation, insulin sensitivity	Skeletal muscle, brown adipose tissue (BAT)	Untrained males and females completed: (1) 90 min steady-state cycling at ~60% VO_2_max or (2) Interval training alternating between 120% and 20% VO_2_max; OR 3 h acute cycling at 40% or 80% VO_2_max	Hypomethylation; dose-dependent ↑ mRNA expression	↑ Mitochondrial density, improved oxidative metabolism, enhanced endurance capacity and insulin sensitivity	[57,68]
*PDKA*	DNA Methylation	Regulates skeletal muscle glucose metabolism	Oxidative skeletal muscle	Same as above (see *PGC-1a* entry)	Hypomethylation; ↑ gene expression at both intensities	Enhanced fat oxidation, reduced reliance on glucose during exercise, improved metabolic flexibility	[57,68]
*MEF2A*	DNA Methylation	Muscle development; regulates *PGC-1α* transcription	Skeletal, cardiac, smooth muscle	Male subjects (physically active) performed a 60 min cycling session at 75 ± 2% VO_2_peak after a 12 h overnight fast	↑ Mitochondrial biogenesis via MEF-PGC-1α interaction; enhanced DNA binding of MEF2 due to HDAC 4/5 dissociation ↑ MEF2 binding	Enhanced mitochondrial function, muscle development, potential protection against T2DM	[89,90]
*FAIM2*	DNA Methylation	Regulates neuronal apoptosis; linked to obesity	Hippocampus	Children categorized as obese or lean based on BMI and weekly physical activity (<150 min/week)	Differential methylation at 7 CpG sites in obese vs. lean children	Potential link to obesity via neural regulation of appetite and stress response	[72]
*HSD11B2*	DNA Methylation	Converts cortisone to cortisol	Muscle, kidney, colon, pancreas, thyroid	Adolescents (wrist accelerometer monitoring); substitution analysis modeling 30 min of vigorous PA replaced with sedentary time	↑ Methylation with increased sedentary time	Potential elevation in cortisol bioavailability → ↑ metabolic risk (e.g., insulin resistance)	[75]
*SOX, GPX*	DNA Methylation	Antioxidant defense, oxidative stress regulation	Liver, lung, kidney, mitochondria, extracellular	Overweight and normal-weight children underwent 24-week aerobic training, followed by 12-week detraining	↑ Gene expression in both overweight and normal weight children	Improved antioxidant defense, reduced oxidative stress, long-term cellular protection	[77]
Histone H3	Histone Modification (Acetylation / Phosphorylation)	Involved in gene activation and response to muscle activity	Skeletal muscle	(1) Healthy males did 10-week resistance training; (2) Elite endurance cyclists completed 8 × 5 min bouts at VO_2_peak with rest intervals	↑ H3 acetylation and phosphorylation post-exercise	Enhanced muscle adaptation, hypertrophy, endurance capacity via gene activation	[86,96]
Histone H3K36	Histone Modification (Acetylation)	Facilitates transcription elongation	Skeletal muscle	Male participants (<2h exercise/week) cycled for 60 min at ~75% VO_2_peak after 12 h fast	↑ H3K36 acetylation in skeletal muscle	Enhanced transcription of exercise-responsive genes → improved muscle adaptation	[87]
Histone H4	Histone Modification (Acetylation)	Regulates muscle growth, metabolism, cytokine production	Skeletal muscle	Schizophrenia patients (male/female mixed) completed aerobic + resistance training 3×/week over 3 months	↑ Acetylation; altered cytokine production	Improved immune response regulation, muscle differentiation, and energy metabolism	[94]
miR-1, miR-133a/b	MicroRNA (miRNA)	Regulate muscle biogenesis, regeneration, and maintenance	Skeletal muscle (myomiRs)	Healthy males (<2h exercise/week) performed 10 days of endurance cycling at ~75% VO_2_peak for 45 min/day	↑ miRNA expression after single session	Promotes muscle repair, growth, and mitochondrial biogenesis	[111]
miR-1, miR-133a, miR-133b, and miR-206	MicroRNA (miRNA)	Regulate muscle biogenesis, regeneration, and maintenance	Skeletal muscle (myomiRs)	Healthy, trained males completed a cycle ergometer (chronic endurance program) 5×/week over 12 weeks	↓ of miR-1, miR-133a, miR-133b, and miR-206 in the human vastus lateralis	Improved endurance capacity, VO_2_max, and insulin sensitivity	[112]
miR-146a, miR-222	MicroRNA (miRNA)	Inflammatory response regulators, linked to chronic disease	Epithelium, monocytes, endothelial cells	Weekly screen time and physical activity assessed over 2 years in underweight and overweigh t children	↑ salivary miRNA expression with increased screen time	Linked to inflammation and possibly increased risk of chronic disease, no PA or BMI correlation	[113]
				**Animal Studies**			
*TFAM*	DNA Methylation	Mitochondrial transcription, replication, and structure	Skeletal muscle	Mice underwent 2 weeks aerobic training (duration and intensity not specified)	↑ *TFAM* expression; increase antioxidant levels	Enhanced mitochondrial function, redox balance, protection against muscle atrophy	[70]
*MEF2A*	DNA Methylation	Regulates muscle development; control *PGC-1α* transcription	Skeletal, cardiac, and smooth muscle	Mice completed moderate aerobic training 5×/week for 3 weeks	↑ *MEF2A* and *PGC-1α* expression	Improved mitochondrial homeostasis, metabolic flexibility, potential T2DM prevention	[71]

Abbrev.: PA: Physical Activity; BMI: Body Mass Index; T2DM: Type 2 Diabetes; *PGC-1α*: Peroxisome proliferator-activated receptor gamma coactivator 1-alpha; *PDK4*: Pyruvate dehydrogenase kinase; *TFAM*: Mitochondrial transcription factor A; *MEF2A*: Myocyte enhancer factor 2A; *FAIM2*: Fas apoptotic inhibitory molecule; *HSD11B2*: Hydroxysteroid (11-beta) dehydrogenase 2. *SOX*: Superoxide; *GPX*: Glutathione Peroxidase.

## Abbreviations

The following abbreviations are used in this manuscript:

FM	Fat Mass
WHO	World Health Organization
T2DM	Type 2 diabetes
LBM	Lean body mass
AMP	Adenosine monophosphate
AMPK	AMP-activated protein kinase
PKA	Protein kinase A
CaMK	Calcium/calmodulin-dependent protein kinase
MAPK	Mitogen-activated protein kinase
PKC	Protein kinase C
mTOR	Mammalian target of rapamycin
PGC-1α	Peroxisome proliferator activated receptor gamma coactivator 1-alpha
CREB	Cyclic AMP response element-binding protein
MEF2-HDAC	Myocyte enhancer factor 2-histone deacetylase
BMI	Body mass index
AVG	Active video games
DMNT	DNA methyltransferase
ROS	Reactive oxygen species
SAM	S-adenosyl methionine
PDK4	Pyruvate dehydrogenase kinase
TFAM	Mitochondrial transcription factor A
MEF2A	Myocyte enhancer factor 2A
FAIM2	Fas apoptotic inhibitory molecule 2
HSB11B2	Hydroxysteroid (11-beta) dehydrogenase 2
SOD	Superoxide dismutase
GPX	Glutathione peroxidase
HATs	Histone acetyl transferase
HDAC	Histone deacetylase
miRNA	MicroRNA
mRNA	Messenger RNA
EWAS	Epigenome-wide association studies
PPARs	Peroxisome proliferator activated receptor
DUSP4	Dual-specificity phosphatase 4
BAIAP3	BAI1 associated protein 3
